# Guideline-concordant cefazolin prophylaxis in penicillin allergy labeled arthroplasty patients: Progress and persistent barriers

**DOI:** 10.1016/j.jcot.2026.103539

**Published:** 2026-06-17

**Authors:** Chiara Anna Giordani, David Dallas-Orr, Shannon Tse, Francesca Anna Giordani, Zachary C. Lum, John P. Meehan, Mauro Giordani

**Affiliations:** a University of California Davis School of Medicine, Sacramento CA, USA; b University of California Davis Department of Orthopaedic Surgery, Sacramento CA, USA; c Università degli Studi di Roma Tor Vergata, Rome, Lazio, Italy

**Keywords:** Arthroplasty, Replacement, Antibiotic prophylaxis, Cefazolin, Cephalosporins, Drug hypersensitivity, Penicillins, Guideline adherence

## Abstract

**Background:**

Cephalosporins are recommended first-line antibiotic prophylaxis for total joint arthroplasty (TJA). However, penicillin allergy labels (PALs), which are often inaccurate, reduce cephalosporin use. In 2022, AAOS released risk-stratification guidelines based on reaction type to safely increase cephalosporin use in PAL patients, categorizing limited hypersensitivity as low-risk, anaphylaxis/angioedema as moderate-risk, and multiorgan hypersensitivity as high-risk. Cefazolin is recommended for all low-risk and some moderate-risk PAL patients, but guideline adherence remains unclear. We evaluated cefazolin administration rates and adverse drug reaction (ADR) incidence in PAL patients before and after guideline dissemination and explored potential barriers to guideline-concordance.

**Methods:**

A retrospective review was conducted on PAL patients undergoing primary TJA (total hip arthroplasty (THA), total knee arthroplasty (TKA), and total shoulder arthroplasty (TSA) at an academic center and two affiliated community hospitals between 2013 and 2024. Revisions were excluded. AAOS risk-stratification guidelines were utilized to classify patients and evaluate guideline-concordance. Date of PAL, antibiotic administration utilized for primary arthroplasty, and ADRs were collected.

**Results:**

Of 172 primary TJAs (49 THAs, 72 TKAs, 51 TSAs) from 130 patients, 92 occurred before and 80 after 2022. Cefazolin administration increased from 64% to 88% (*p = 0.001*) in low-risk and 40% to 64% (*p = 0.281*) in moderate-risk patients. The main alternative was vancomycin with clindamycin (21%). Mean time from PAL to surgery was 9.9 years. No ADRs occurred with any antibiotic. Among 36 patients undergoing ≥2 TJAs, 72% initially received cefazolin, and 50% who initially received alternatives subsequently received cefazolin without ADRs.

**Conclusion:**

Cefazolin administration increased after guideline release, yet hesitancy persisted even in low-risk patients. All PAL patients who received cefazolin tolerated it. Documented PALs continue reducing the likelihood of receiving first-line antibiotic recommendations. PAL documentation quality and timing were identified as potential barriers to guideline-concordance. Targeted initiatives may ensure appropriate, evidence-based TJA prophylaxis.

## 1 Introduction

The American Academy of Orthopaedic Surgery (AAOS) recommends cefazolin, a first-generation cephalosporin, as the first-line perioperative antibiotic for patients undergoing total joint arthroplasty (TJA).^1^ This aligns with the International Consensus Meeting on Musculoskeletal Infection (ICM) guidelines, which recommend a cephalosporin against both gram-positive and gram-negative bacteria^2^ for TJA. Deviating from these standards has significant clinical consequences; it has been well established that receiving non-cephalosporin antibiotic alternatives increases the risk for prosthetic joint infection (PJI).^3^

Despite the clear benefits of cefazolin in TJA, its use is frequently curtailed in practice by a penicillin allergy label (PAL). Penicillin is the most commonly reported drug allergy. 4, 5, 6 However, the majority of PALs are inaccurate, with studies indicating that up to 90% of patients with a PAL do not have a true type 1 hypersensitivity reaction.^4^ Numerous studies demonstrate that cefazolin prophylaxis should not be immediately contraindicated in TJA patients with a PAL ^3^^,^^7^, ^8^, ^9^, and AAOS recommends alternative antibiotics only for patients with true IgE-mediated allergic reactions to penicillin.^1^

To bridge the gap between labeled allergies and safe clinical practice, institutions have utilized various antimicrobial stewardship measures for TJA patients with PAL, including drug allergy clinic (DAC) referrals,^10^ administration of cefazolin challenge doses,^7^^,^^11^ and risk stratification protocols.^12^^,^^13^ In 2022, the AAOS introduced a formalized risk-stratification tool that categorizes patients into low, moderate, or high-risk groups, based on the severity of their prior adverse drug reaction (ADR) to penicillin.^4^ The recommendation is to administer cefazolin to low-risk patients and clindamycin or vancomycin to high-risk patients.^12^ Moderate-risk patients can consider allergy testing referral in the elective setting or receive clindamycin or vancomycin.^12^ While this tool provides a clear pathway for PAL patients, it remains unclear how effectively these guidelines have been integrated into surgical practice.

The primary purpose of this study was to evaluate concordance with the 2022 AAOS risk-stratification guidelines by comparing cephalosporin use in PAL patients before and after their release. Secondarily, we assessed incidence of adverse drug reactions (ADRs) and explored potential barriers to guideline-concordant care.

## 2 Methods

Following institutional review board exemption, a retrospective review of all adult patients with a documented PAL who underwent total knee arthroplasty (TKA), total hip arthroplasty (THA), or reverse or anatomic shoulder arthroplasty (TSA) at an academic center and two affiliated community hospitals between 2013 and 2024 was conducted. Patients over 18 years of age, including those undergoing more than one primary TJA, were analyzed in the specified study period. Patients without a formally documented PAL and those undergoing revision surgery were excluded.

Electronic medical records were reviewed to collect patient demographics, including age, sex, procedure type, and date of surgery. Surgery date was used to group patients into pre-2022 and post-2022 cohorts. The date PAL was documented was noted, and the mean time in years from documented PAL to primary TJA was calculated. Anesthesia and postoperative reports were utilized to record antibiotics given and ADRs in the perioperative period. Because some PAL patients underwent multiple primary TJAs with different antibiotic regimens, each primary TJA was evaluated as an independent surgical event.

Documented reactions to penicillin were collected for grouping of cohorts into low, moderate, or high-risk categories per the AAOS risk-stratification tool.^12^ Low-risk PAL patients included those with documented side effects or intolerances: gastrointestinal symptoms, headache, muscle aches, or psychiatric disturbances), limited hypersensitivity reactions (self-limited cutaneous rash, urticaria >5 years ago, or itching), or nonspecific reaction (unknown reaction, remote childhood reaction). Patients with documented disseminated hypersensitivity or anaphylaxis (swelling of face or throat, angioedema, difficulty breathing, or urticaria <5 years ago) were classified as moderate-risk. High-risk PAL patients had documented Stevens-Johnson syndrome or toxic epidermal necrolysis (diffuse ulceration, blistering, or pustulosis) or multiorgan hypersensitivity response (history of kidney or liver injury).

Chi-square tests were utilized to compare cefazolin administration rates and ADRs in the pre- and post-2022 cohorts. For the moderate-risk group and for the subgroup analyses of patients undergoing multiple procedures, Fisher's exact test was utilized to compare antibiotic administration patterns due to small expected cell counts. A p-value <0.05 was considered statistically significant. SPSS (Version 29, IBM, Chicago, IL, USA) was utilized to conduct statistical analyses.

## 3 Results

The final cohort meeting inclusion criteria consisted of 130 PAL patients, with 41 men (31.5%) and 89 women (68.5%). The mean age at time of surgery was 70 years (range, 20 –93). The mean (± standard deviation) time in years from documented PAL to primary TJA was 9.9 ± 6.6 years. There were 172 primary TJAs, including 72 TKAs (41.9%), 49 THAs (28.5%), 18 anatomic TSAs (10.5%), and 33 reverse TSAs (19.2%). There were 92 (53.5%) TJAs that occurred before 2022 and 80 (46.5%) TJAs occurred after the release of the 2022 AAOS guidelines. A total of 12 arthroplasty surgeons performed surgeries on this cohort.

When using the AAOS risk stratification tool, there were 135 primary TJAs categorized as low-risk, with rash/hives/itching as the most common documented reaction (Table 1). The antibiotics used for surgical prophylaxis for each TJA have been summarized in Table 2. Cefazolin administration increased significantly in low-risk patients after the 2022 AAOS guideline release (64% to 88%; χ^2^ = 10.80, *p = 0.001*). Among moderate-risk patients, cefazolin use did not increase significantly (40% to 64%, Fisher's exact, *p = 0.281*). There was only one high-risk case in this study, which occurred after 2022; given n = 1, no conclusions can be drawn regarding this subgroup. When cefazolin was not administered, dual coverage with clindamycin and vancomycin was the most common alternative antibiotic (Table 2). No ADRs resulted from alternative antibiotic use (Table 3).

**Table 1:** Penicillin allergy label documented reaction severity.

AAOS Severity	Number of Patients, n	Most Common Reaction	Patients with Most Common Reaction, n (%)
Low	103	Rash/Hives/Itching	68 (66%)
Moderate	26	Anaphylaxis	14 (54%)
High	1	Stevens-Johnson Syndrome	1 (100%)
Total	130		

*Abbreviations:* AAOS = American Academy of Orthopaedic Surgery.

**Table 2:** Antibiotic administration by surgical procedure type.

Surgery Type	Cefazolin Single, n (%)	Cefazolin + Vancomycin, n (%)	Vancomycin Single, n (%)	Vancomycin + Clindamycin, n (%)	Vancomycin + Gentamicin, n (%)	Clindamycin Single, n (%)	Gentamicin Single, n (%)	Total, n
TKA	14 (19%)	44 (61%)	1 (1%)	10 (14%)	1 (1%)	2 (3%)	0 (0%)	72
THA	2 (4%)	36 (74%)	0 (0%)	7 (14%)	1 (2%)	2 (4%)	1 (2%)	49
TSA	4 (8%)	21 (41%)	3 (6%)	19 (37%)	1 (2%)	3 (6%)	0 (0%)	51
Total	20 (12%)	101 (59%)	4 (2%)	36 (21%)	3 (2%)	7 (4%)	1 (1%)	172

*Note:* TSA includes anatomic and reverse total shoulder arthroplasty.

*Abbreviations:* THA = total hip arthroplasty; TKA = total knee arthroplasty; TSA = total shoulder arthroplasty.

**Table 3:** Cefazolin administration rates and ADR reaction rates of penicillin allergy label patients according to each AAOS risk severity.

AAOS Severity	Pre-2022			Post-2022		
	Primary TJAs, n	Cefazolin Rate, n (%)	ADR Rate, n (%)	Primary TJAs, n	Cefazolin Rate, n (%)	ADR Rate, n (%)
Low	67	43 (64%)	0 (0%)	68	60 (88%)^a^	0 (0%)
Moderate	25	10 (40%)	0 (0%)	11	7 (64%)	0 (0%)
High	0	N/A	N/A	1	1 (100%)^c^	0 (0%)
Total	92	53 (58%)		80	68 (85%)^b^	

*Abbreviations:* AAOS = American Academy of Orthopaedic Surgery; TJA = total joint arthroplasty; ADR = adverse drug reaction; N/A = not applicable.

a *p = 0.001* vs pre-2022 (chi-square).

b *p < 0.001* vs pre-2022 (chi-square); Moderate-risk: *p = 0.281* (Fisher's exact).

c n = 1, no conclusions can be drawn for the high-risk subgroup.

There were 36 PAL patients who underwent two or more TJAs between 2013 and 2024 (Table 4). Of these patients, 26 (72%) received cefazolin for their first procedure. Half of the patients who initially received alternative antibiotics subsequently received cefazolin for their second or third surgery, without ADRs. Among patients who went on to receive cefazolin for their subsequent surgery, 80% were classified as low-risk. All 26 patients who received cefazolin for their first procedure also received it again for later surgeries, compared with 5 of 10 patients who initially received an alternative antibiotic (Fisher's exact test, *p = 0.001*).

**Table 4:** Sub-analysis of antibiotics administered in patients undergoing multiple joint arthroplasties.

AAOS Severity	Total Patients Undergoing Multiple TJAs, n	Received Cefazolin for 1st Surgery, n (%)	Received Alternate antibiotic for 1st Surgery, n (%)	Received Cefazolin for 2nd or 3rd Surgery after Alternate, n (%)
Low	28	22 (78.5%)	6 (21.4%)	4 (66.7%)
Moderate	8	4 (50.0%)	4 (50.0%)	1 (25.0%)
High	0	0 (0.0%)	0 (0.0%)	0 (0.0%)
Total	36	26 (72.2%)	10 (27.8%)	5 (50%)

*Abbreviations:* AAOS = American Academy of Orthopaedic Surgery; TJA = total joint arthroplasty.

## 4 Discussion

This study evaluated whether the 2022 AAOS guidelines were associated with changes in cefazolin administration in PAL patients and whether ADRs occurred in those receiving first-line recommendations. The findings indicate ongoing hesitancy in administering cefazolin to low-risk and moderate-risk PAL patients, despite updated recommendations. While all low-risk patients should have received cefazolin, only 88% of the cohort was guideline-concordant. This rate was 64% in the moderate-risk group. While the moderate-risk group did not have a statistically significant increase in cefazolin administration post-2022, thus pointing toward marked guideline discordance, these results should be interpreted in the context of this sub-group's smaller sample size.

Potential reasons for reluctance include historical concerns about cephalosporin cross-reactivity with penicillin, the perceived severity of documented adverse reactions, and poor PAL documentation. Historical evidence demonstrated 10% cross-reactivity between cephalosporins with penicillin ^12^^,^^14^, ^15^, ^16^; however, there is an increasing body of literature refuting these early studies ^12^^,^^17^, ^18^, ^19^ attributing cross-reactivity to trace contaminants from manufacturing of cephalosporins prior to the 1980s.^12^^,^^20^ The cross-reactivity rate in patients with a true penicillin allergy has been determined to be 1% when using a first-generation cephalosporin such as cefazolin, or a cephalosporin with a similar R1 side chain as penicillin.^21^ While every patient in this cohort carried a PAL in their medical record, none developed ADRs after receiving cefazolin. Caution is warranted in interpreting this finding: the retrospective design relies on chart documentation and may reflect under-capture. These findings nonetheless suggest that outdated concerns about cross-reactivity may contribute to the continued avoidance of cefazolin in PAL patients.

Perceived severity of documented allergic reactions could also be contributing to use of second-line antibiotics in low- and moderate-risk patients. Despite AAOS recommendations for risk-based stratification, skin testing and preoperative referral to a drug allergy clinic were rarely utilized in this cohort, likely due to time-constraints and a preference for avoiding surgical delays when alternative antibiotic combinations were available.^22^^,^^23^ Consistent with the AAOS guidelines, clindamycin with vancomycin was the most used alternative antibiotic to cefazolin for PAL patients at these institutions. None of the patients with a documented history of anaphylaxis underwent preoperative skin testing, and only one patient in the entire cohort received a skin test. This patient received a TJA in 2023, carried a low-risk reaction (rash) in their chart, tested negative on skin test, and subsequently received cefazolin without complications. Given that anaphylaxis occurs in 0.02-0.04% of true penicillin allergic patients,^24^ and that the recent guidelines recommend skin testing or referral to drug allergy clinic for moderate-risk PAL patients, integrating standardized testing or challenge doses^25^ could help reduce unnecessary cefazolin avoidance and improve patient safety.

Penicillin allergies are often over-documented, potentially leading to unnecessary avoidance of beta-lactam antibiotics.^22^ Studies have shown that poor allergy records increase likelihood of unnecessary second-line antibiotic use, elevating risks of surgical site infections and poorer outcomes.^3^ It has been hypothesized that overdocumentation is due to childhood diagnoses, misclassification of non-allergic events, or labeling reactions that are low-risk for immediate hypersensitivity.^20^ Our analysis revealed substantial variation in penicillin allergy documentation (Supplemental Table 1). Of the 130 patients with documented PAL, 25 (19.2%) had no specific reaction detailed in their medical record beyond “penicillin allergy”. Only 13 patients (10%) had documentation of a childhood reaction: 10 patients specified that the allergy occurred as “child”, 1 as “young boy”, and 2 as “infant” (Supplemental Table 1). Critically, all documented penicillin allergies in our cohort were patient-reported, with only one patient having undergone formal allergy testing. Initially, our study aimed to distinguish cefazolin administration rates between self-reported versus allergist-confirmed PAL, childhood versus adult-onset allergy, and true IgE-mediated reaction in the context of the AAOS guidelines. However, the near universal reliance on self-reported allergy history (99.2%) without verification prevented these comparisons and introduced misclassification bias. We identified a need for more accurate and up-to-date documentation of penicillin allergy history, as true reactions could have consequences for patients.

Notably, our study's findings suggest an association between cefazolin use in the first surgery and subsequent cefazolin use in PAL patients undergoing multiple TJAs. In our cohort, all 26 patients who received cefazolin for their first procedure also received it again for later surgeries, compared with 5 of 10 patients who initially received an alternative antibiotic (Fisher's exact test, *p = 0.001*). While statistically significant, this observation is based on a small subset of our cohort (n = 36 patients undergoing multiple procedures), which limits the generalizability of this finding and may also reflect temporal confounders. Despite successful administration of cefazolin, these patients retained their PAL in their medical record without an addended specification of no cross-reactivity with cefazolin. Among patients who did not receive cefazolin for their first surgery, 50% received it for their second or third TJA without adverse effects. This suggests that they may have potentially safely received cefazolin earlier or that their allergy status changed over time. Given that penicillin sensitivity declines naturally, the authors recommend careful documentation of allergy onset date and re-evaluation over time. Research has indicated that in patients with true IgE-mediated reactions to penicillin, 50% lose sensitivity after 5 years, and 80% lose sensitivity after 10 years.^6^^,^^24^ In this cohort, the mean number of years from penicillin allergy label to date of the primary TJA was 9.9 years. The clinical implications of this temporal gap are substantial: even if every PAL patient in our cohort had initially experienced a true acute allergy, which studies suggest occurs in less than 10% those carrying a PAL, approximately 80% would have lost sensitivity by the time of their arthroplasty.^4^^,^^24^ To illustrate the potential magnitude of this effect, extrapolation estimates that as few as 2-3% of our cohort (approximately 3 of 130 patients) likely retained a clinically significant penicillin allergy at the time of surgery. Despite this, 42 patients (32.3%) in the post-2022 cohort received alternative antibiotics rather than first-line cefazolin, representing a substantial number of patients who may have been unnecessarily exposed to inferior perioperative prophylactic regimens. While not a study-derived estimate, this contextual approximation underscores both the natural history of penicillin sensitivity and the critical need for systematic PAL reassessment, particularly when significant time has elapsed since the initial allergic event.

Previous studies demonstrated that multifaceted interventions effectively and safely improve guideline-concordance while decreasing costs to hospital systems. Jones et al. implemented an updated institutional guideline that differentiated between severe and non-severe penicillin allergies; notably, non-severe allergies included those with a remote history (>10 years) of penicillin exposure with an unknown reaction.^26^ A key component of their antibiotic stewardship intervention included an updated institutional guideline that helped differentiate between severe and non-severe penicillin allergies; notably, non-severe allergies included those with skin rash, pruritus, urticaria, local swelling, intolerance (nausea, vomiting, diarrhea, headache), or a remote history (>10 years) of penicillin exposure with an unknown reaction. Additional stewardship interventions included a preoperative allergy assessment, a standard presurgical screening order set, and an orthopaedic physician champion who provided education to peers to increase compliance.^26^ Following implementation of these interventions, appropriate antibiotic use increased significantly from 54% to 91% with no adverse drug effects.^26^ Pagani et al. found that routine preoperative testing in PAL patients undergoing elective TJA both increased the number of patients eligible to safely receive cefazolin and proved cost-effective relative to the hospital burden of prosthetic joint infections associated with non-cefazolin use.^27^ This contextualizes the cost of PJI, guideline-concordance, and antibiotic stewardship initiatives on a system-level.

To translate these findings into practice, we propose a structured preoperative PAL assessment pathway for TJA patients in concordance with AAOS guidelines. PAL patients would benefit from having their documented reaction reviewed. Low-risk patients (non-specific or remote/mild cutaneous reaction) should receive cefazolin as standard prophylaxis with no further workup required. Moderate-risk patients (documented anaphylaxis, angioedema, or urticaria within 5 years) would benefit from referral to a drug allergy clinic for preoperative skin testing or graded challenge when scheduling permits; if time constraints preclude testing, clindamycin with vancomycin may be used with documentation of the rationale. High-risk patients (Stevens-Johnson syndrome or multiorgan hypersensitivity) should receive alternative antibiotics. Most importantly, all allergy records should be updated after each surgical encounter to reflect whether cefazolin was tolerated in the context of PAL, creating a cumulative clinical record that reduces unnecessary avoidance in future procedures.

This study is not without limitations. This evaluation occurs just 2 years after the 2022 AAOS recommendations, and implementing changes to protocols and practices may have been challenging within this short timeframe. Furthermore, the retrospective design does not adjust for temporal confounders, which limits causal attribution of the observed usage of cefazolin to the 2022 AAOS guidelines. The absence of ADRs is notable, but should be interpreted cautiously given the sample sizes in this study. Findings should not be interpreted as definitive safety conclusions, and further research is needed to assess cefazolin administration trends over the next 5-10 years with adjustment for concurrent factors. This includes assessing ADRs in high-risk PAL patients, which were severely underrepresented. With only one high-risk patient in our cohort, no conclusions can be drawn regarding cefazolin administration or ADR risk in this group. Treating each TJA as an independent surgical event, while consistent with the study's perioperative focus, may introduce within-patient clustering bias. Mixed-effects models were not applied given the smaller sample sizes involved in subgroup analyses, and findings should be interpreted with this limitation in mind. Finally, reliance on self-reported allergy history introduced misclassification bias and prevented planned subgroup comparisons.

## 5 Conclusions

This study evaluated real-world adherence to the 2022 AAOS risk-stratification guidelines and found persistence of an evidence-to-practice gap in cefazolin administration to PAL arthroplasty patients. While no PAL patients who received cefazolin had adverse effects, the authors emphasize that patients may still have independent allergies to cephalosporins or may have ADRs. Therefore, PAL should not be disregarded when planning cephalosporin use prior to TJA. Although the 2022 AAOS risk stratification guidelines have improved first-line antibiotic prophylaxis in TJA patients carrying a PAL in this cohort, concordance remains lacking. The authors recommend improving PAL documentation and preoperative planning for PAL patients to ensure evidence-based prophylaxis and minimize potential for PJI.

## Informed consent and patient details

This was a retrospective cohort study reviewed by the Institutional Review Board (IRB) and exempt.

## Author credit statement

CAG: conceptualization, formal analysis, investigation, project administration, visualization, writing- original draft, writing – review & editing.

DDO: data curation, formal analysis, writing – original draft.

ST: data curation, writing – review & editing.

FAG: visualization, writing – original draft, writing – review & editing.

ZCL: methodology, resources, supervision, writing – review & editing.

JPM: resources, supervision, writing – review & editing.

MG: methodology, resources, supervision, conceptualization, investigation, project administration, writing – review & editing.

## Ethical statement

This project was IRB reviewed and exempt. Please review the details below:

• Name of the IRB or Ethics Committee: UC Davis IRB Administration, Davis, CA
• Letter Number: UC Davis Committee B, Davis, CA Package #: 2122525-1
• Board Action: Exempt, Date: 12/13/2023

## Funding statement

This research did not receive any specific grant from funding agencies in the public, commercial or not-for-profit sectors.

## Declaration of competing interest

The authors declare the following financial interests/personal relationships which may be considered as potential competing interests: Mauro Giordani reports a relationship with California Orthopaedic Association that includes: board membership. Mauro Giordani reports a relationship with American Academy of Orthopaedic Surgeons that includes: board membership. Zachary Lum reports a relationship with American Association of Hip and Knee Surgeons that includes: board membership. Zachary Lum reports a relationship with JAOAO that includes: non-financial support. ZCL is an Editoral Board Member/Edit-in-Chief for Journal of Clinical Orthopaedics and Trauma and was not involved in the editorial review or the decision to publish this article. All authors declare that there are no competing interests.
