# Supplementary material for: Guideline-concordant cefazolin prophylaxis in penicillin allergy labeled arthroplasty patients: Progress and persistent barriers
Source: J Clin Orthop Trauma. 2026 Jun 17;81:103539. doi: 10.1016/j.jcot.2026.103539 (PMC13326071; doi:10.1016/j.jcot.2026.103539)
Supplement: Supplementary file 1 [file mmc1.docx]

# **Supplemental Table 1: Detailed Penicillin Allergy Documentation and Reaction Types**

| **Category** | **n (% of 130 patients)** |
| --- | --- |
| **Reaction Specificity** |  |
| Specific reaction documented | 105 (80.8%) |
| No reaction specified | 25 (19.2%) |
|  |  |
| **Timing of Allergy** |  |
| Childhood-onset documented* | 13 (10.0%) |
| With specific reaction details | 6 (46.2% of childhood) |
| Without specific reaction details† | 7 (53.8% of childhood) |
|  |  |
| **Documented Reaction Types** |  |
| **Cutaneous/Localized** |  |
| Rash/hives/itching (isolated or combined) | 68 (52.3%) |
| Localized swelling | 6 (4.6%) |
| Stevens-Johnson syndrome (skin peeling) | 1 (0.8%) |
|  |  |
| **Gastrointestinal** |  |
| Diarrhea alone | 6 (4.6%) |
| Nausea/vomiting alone | 3 (2.3%) |
| Combined GI symptoms | 3 (2.3%) |
|  |  |
| **Systemic/Severe Reactions** |  |
| Anaphylaxis documented | 14 (10.8%) |
| Angioedema | 1 (0.8%) |
| Shortness of breath alone | 8 (6.2%) |
| Throat swelling/closure | 3 (2.3%) |
|  |  |
| **Other Reactions** |  |
| Neuropsychiatric (hallucinations) | 2 (1.5%) |
| Suspected thrombocytopenia | 1 (0.8%) |
| Palpitations (with cutaneous symptoms) | 1 (0.8%) |
|  |  |
| **Allergy Verification Status** |  |
| Self-reported only | 129 (99.2%) |
| Formal allergy testing performed | 1 (0.8%) |
| Test result: negative | 1 (100% of tested) |
| *Thirteen patients had documentation of childhood-onset allergy: 10 documented as "child," 1 as "young boy," and 2 as "infant"  †Seven of the 13 childhood-onset allergies had no specific reaction documented and are included in the 25 patients with "no reaction specified." The remaining 6 childhood-onset patients had documented reactions including rash (n=2), hives (n=1), anaphylaxis/rash/angioedema (n=1), and hallucinations (n=2)  **Note:** Some patients had multiple symptoms documented (e.g., hives with shortness of breath); such cases were categorized based on the most severe symptom per AAOS risk stratification guidelines. Percentages are based on 130 unique patients with documented penicillin allergy labels.  **Abbreviations:** AAOS = American Academy of Orthopaedic Surgeons | |
